# 3-Fluoro-4-(4-hy­droxy­phen­oxy)benzonitrile

**DOI:** 10.1107/S1600536810024360

**Published:** 2010-06-30

**Authors:** Mei Zheng, Jianfeng Wang, Jixu Zhang, Shuping Luo

**Affiliations:** a Hangzhou Huadong Medicine Group Biotechnology Institute Co. Ltd, Hangzhou 310015, People’s Republic of China; b State Key Laboratory Breeding Base of Green Chemistry–Synthesis Technology, Zhejiang University of Technology, Hangzhou 310014, People’s Republic of China

## Abstract

The title compound, C_13_H_8_FNO_2_, was synthesized from 3,4-difluoro­benzonitrile and hydro­quinone. The dihedral angle between the two aromatic rings is 70.9 (2)°. In the crystal structure, mol­ecules are linked by O—H⋯N hydrogen bonds, forming zigzag chains.

## Related literature

For the herbicidal actvity of hydro­quinone derivatives, see: Bao *et al.* (2007 ▶); Liu (2002 ▶). For related structures, see: Sørensen *et al.* (2009 ▶); Luo *et al.* (2009 ▶); Zhang *et al.* (2009 ▶).
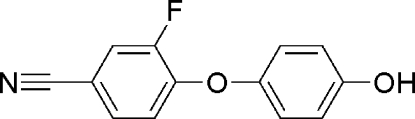


## Experimental

### 

#### Crystal data

C_13_H_8_FNO_2_
*M* *_r_* = 229.20
Orthorhombic, 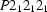

*a* = 6.1932 (4) Å
*b* = 8.8109 (5) Å
*c* = 20.5269 (12) Å
*V* = 1120.11 (12) Å^3^
*Z* = 4
Mo *K*α radiation
μ = 0.10 mm^−1^
*T* = 295 K
0.39 × 0.31 × 0.22 mm

#### Data collection

Rigaku R-AXIS RAPID diffractometer
Absorption correction: multi-scan (*ABSCOR*; Higashi, 1995 ▶) *T*_min_ = 0.959, *T*_max_ = 0.976
10999 measured reflections
1498 independent reflections
928 reflections with *I* > 2σ(*I*)
*R*_int_ = 0.032

#### Refinement

*R*[*F* ^2^ > 2σ(*F* ^2^)] = 0.037
*wR*(*F* ^2^) = 0.117
*S* = 1.01
1498 reflections
156 parameters
H-atom parameters constrained
Δρ_max_ = 0.17 e Å^−3^
Δρ_min_ = −0.15 e Å^−3^

### 

Data collection: *PROCESS-AUTO* (Rigaku, 2006 ▶); cell refinement: *PROCESS-AUTO*; data reduction: *CrystalStructure* (Rigaku/MSC, 2007 ▶); program(s) used to solve structure: *SHELXS97* (Sheldrick, 2008 ▶); program(s) used to refine structure: *SHELXL97* (Sheldrick, 2008 ▶); molecular graphics: *ORTEP-3 for Windows* (Farrugia,1997 ▶); software used to prepare material for publication: *WinGX* (Farrugia, 1999 ▶).

## Supplementary Material

Crystal structure: contains datablocks I, global. DOI: 10.1107/S1600536810024360/im2202sup1.cif

Structure factors: contains datablocks I. DOI: 10.1107/S1600536810024360/im2202Isup2.hkl

Additional supplementary materials:  crystallographic information; 3D view; checkCIF report

## Figures and Tables

**Table 1:** Hydrogen-bond geometry (Å, °)

*D*—H⋯*A*	*D*—H	H⋯*A*	*D*⋯*A*	*D*—H⋯*A*
O2—H201⋯N1^i^	0.82	2.03	2.839 (4)	168

Symmetry code: (i) 
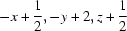
.

**Fig. 1.**
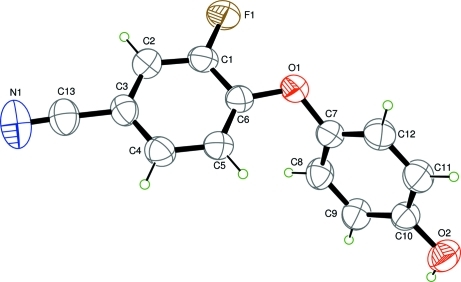
Molecular structure of title compound, with the atomic labeling scheme. Displacement ellipsoids are drawn at the 50% probability level.

**Fig. 2.**
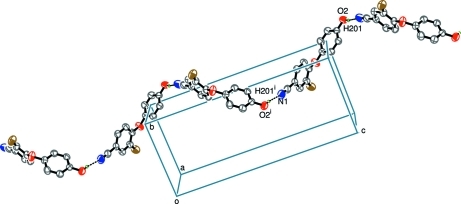
A partial packing diagram of title compound. Hydrogen bonds are shown as dashed lines. [Symmetry code: (i) -x+1/2, -y+2, z+1/2].

## supplementary crystallographic information

### Comment

Hydroquinone derivatives are important intermediates of herbicide synthesis and have therefore received growing attention recently (Liu, 2002; Bao *et al.*, 2007). Several hydroquinone derivatives were synthesized and investigated by X-ray diffraction in our laboratory. 4-(4-Cyano-2-fluoro-phenoxy)-phenol was obtained reacting hydroquinone and 3,4-difluorobenzonitrile and it's molecular structure is shown in Fig.1.

As it is expected substituents at both aromatic rings are coplanar with repect to the aromatic planes. The dihedral angle between the two planes is 70.66°. The molecule is bent with a C6—O1—C7 angle of 118.0 (2)°. The crystal structure is determined by intermolecular O—H···N interactions. The resulting supramolecular chains of the title compound showing H-bridge interactions is shown in Fig.2.

### Experimental

A DMSO (10 ml) solution of hydroquinone (0.0012 mol) and NaOH (0.0024 mol) was stirred at room temperature for 5 h. Then the mixture was heated to 80°C and 3,4-difluorobenzonitrile (0.001 mol) was added dropwise and stirred for 10 h. Then the mixture was washed with water (30 ml) and extracted with ethyl acetate (three times). The organic solvent was removed under reduced pressure and the resulting crude product was purified by silica gel chromatography (pentane: ethyl acetate mixtures, yield 86%). Single crystals were obtained by slow evaporation of ethyl acetate at room temperature.

### Refinement

In the absence of significant anomalous dispersion effects, Friedel pairs were averaged. H atoms were placed in calculated positions with C—H = 0.98 Å (sp), C—H = 0.97 Å (sp^2^), C—H = 0.93 Å (aromatic). All H atoms were included in the final cycles of refinement using a riding model, with U_iso_(H)=1.2U_eq_ of the respective carrier atoms.

### Crystal data

**Table:**

C_13_H_8_FNO_2_	*F*(000) = 472
*M**_r_* = 229.20	*D*_x_ = 1.359 Mg m^−^^3^
Orthorhombic, *P*2_1_2_1_2_1_	Mo *K*α radiation, λ = 0.71073 Å
Hall symbol: P 2ac 2ab	Cell parameters from 6842 reflections
*a* = 6.1932 (4) Å	θ = 3.0–27.4°
*b* = 8.8109 (5) Å	µ = 0.10 mm^−^^1^
*c* = 20.5269 (12) Å	*T* = 295 K
*V* = 1120.11 (12) Å^3^	Chunk, colorless
*Z* = 4	0.39 × 0.31 × 0.22 mm

### Data collection

**Table:**

Rigaku R-AXIS RAPID diffractometer	1498 independent reflections
Radiation source: rolling anode	928 reflections with *I* > 2σ(*I*)
graphite	*R*_int_ = 0.032
Detector resolution: 10.00 pixels mm^-1^	θ_max_ = 27.4°, θ_min_ = 3.1°
ω scans	*h* = −8→8
Absorption correction: multi-scan (*ABSCOR*; Higashi, 1995)	*k* = −11→11
*T*_min_ = 0.959, *T*_max_ = 0.976	*l* = −26→26
10999 measured reflections	

### Refinement

**Table:**

Refinement on *F*^2^	Secondary atom site location: difference Fourier map
Least-squares matrix: full	Hydrogen site location: inferred from neighbouring sites
*R*[*F*^2^ > 2σ(*F*^2^)] = 0.037	H-atom parameters constrained
*wR*(*F*^2^) = 0.117	*w* = 1/[σ^2^(*F*_o_^2^) + (0.0487*P*)^2^ + 0.2503*P*] where *P* = (*F*_o_^2^ + 2*F*_c_^2^)/3
*S* = 1.01	(Δ/σ)_max_ < 0.001
1498 reflections	Δρ_max_ = 0.17 e Å^−^^3^
156 parameters	Δρ_min_ = −0.15 e Å^−^^3^
0 restraints	Extinction correction: *SHELXL97* (Sheldrick, 2008), Fc^*^=kFc[1+0.001xFc^2^λ^3^/sin(2θ)]^-1/4^
Primary atom site location: structure-invariant direct methods	Extinction coefficient: 0.031 (5)

### Special details

**Table:**

Geometry. All esds (except the esd in the dihedral angle between two l.s. planes) are estimated using the full covariance matrix. The cell esds are taken into account individually in the estimation of esds in distances, angles and torsion angles; correlations between esds in cell parameters are only used when they are defined by crystal symmetry. An approximate (isotropic) treatment of cell esds is used for estimating esds involving l.s. planes.
Refinement. Refinement of *F*^2^ against ALL reflections. The weighted *R*-factor wR and goodness of fit *S* are based on *F*^2^, conventional *R*-factors *R* are based on *F*, with *F* set to zero for negative *F*^2^. The threshold expression of *F*^2^ > σ(*F*^2^) is used only for calculating *R*-factors(gt) etc. and is not relevant to the choice of reflections for refinement. *R*-factors based on *F*^2^ are statistically about twice as large as those based on *F*, and *R*- factors based on ALL data will be even larger.

### Fractional atomic coordinates and isotropic or equivalent isotropic displacement parameters (Å^2^)

**Table:**

	*x*	*y*	*z*	*U*_iso_*/*U*_eq_	
O1	0.8260 (4)	0.8262 (3)	0.91021 (10)	0.0842 (7)	
F1	0.7477 (4)	0.5751 (2)	0.84401 (10)	0.0994 (7)	
C3	0.2965 (5)	0.7967 (4)	0.78841 (13)	0.0647 (8)	
C13	0.1155 (6)	0.7849 (4)	0.74589 (16)	0.0797 (10)	
C7	0.8277 (5)	0.9255 (4)	0.96363 (13)	0.0662 (8)	
O2	0.8846 (4)	1.2010 (3)	1.12504 (11)	0.0890 (8)	
H201	0.7775	1.1944	1.1484	0.134*	
C4	0.3341 (5)	0.9302 (4)	0.82157 (14)	0.0706 (8)	
H4	0.2411	1.0120	0.8158	0.085*	
C10	0.8605 (5)	1.1076 (3)	1.07184 (13)	0.0647 (8)	
C1	0.6082 (5)	0.6918 (4)	0.83712 (14)	0.0689 (8)	
C8	0.6707 (5)	0.9204 (4)	1.01045 (14)	0.0720 (8)	
H8	0.5543	0.8546	1.0059	0.086*	
C6	0.6460 (5)	0.8233 (4)	0.87182 (13)	0.0648 (8)	
C2	0.4369 (5)	0.6748 (4)	0.79642 (14)	0.0712 (8)	
H2	0.4141	0.5839	0.7744	0.085*	
C9	0.6853 (5)	1.0130 (4)	1.06440 (14)	0.0718 (9)	
H9	0.5769	1.0115	1.0958	0.086*	
C11	1.0188 (6)	1.1111 (4)	1.02514 (15)	0.0789 (10)	
H11	1.1376	1.1745	1.0301	0.095*	
C12	1.0011 (6)	1.0202 (4)	0.97087 (15)	0.0805 (10)	
H12	1.1076	1.0231	0.9390	0.097*	
C5	0.5080 (6)	0.9438 (4)	0.86314 (14)	0.0710 (9)	
H5	0.5319	1.0344	0.8853	0.085*	
N1	−0.0310 (6)	0.7800 (4)	0.71268 (15)	0.1078 (12)	

### Atomic displacement parameters (Å^2^)

**Table:**

	*U*^11^	*U*^22^	*U*^33^	*U*^12^	*U*^13^	*U*^23^
O1	0.0678 (14)	0.1042 (16)	0.0805 (13)	0.0258 (14)	−0.0173 (12)	−0.0286 (13)
F1	0.0986 (16)	0.0867 (12)	0.1129 (14)	0.0398 (13)	−0.0214 (12)	−0.0218 (12)
C3	0.0581 (18)	0.083 (2)	0.0534 (14)	−0.0009 (17)	−0.0006 (13)	0.0069 (16)
C13	0.076 (2)	0.092 (2)	0.0710 (19)	−0.009 (2)	−0.0057 (19)	0.0122 (18)
C7	0.0610 (18)	0.0736 (17)	0.0642 (15)	0.0089 (17)	−0.0031 (16)	−0.0066 (15)
O2	0.0893 (19)	0.0916 (15)	0.0861 (15)	−0.0181 (15)	0.0185 (13)	−0.0244 (14)
C4	0.069 (2)	0.0754 (19)	0.0671 (16)	0.0133 (18)	−0.0032 (17)	0.0033 (16)
C10	0.066 (2)	0.0648 (17)	0.0632 (15)	−0.0042 (16)	0.0052 (16)	−0.0025 (14)
C1	0.068 (2)	0.0692 (18)	0.0697 (17)	0.0159 (17)	0.0009 (16)	−0.0051 (17)
C8	0.065 (2)	0.0776 (18)	0.0733 (17)	−0.0135 (18)	0.0016 (17)	−0.0006 (17)
C6	0.0592 (19)	0.0778 (18)	0.0572 (15)	0.0090 (17)	0.0002 (14)	−0.0069 (15)
C2	0.072 (2)	0.0751 (19)	0.0665 (17)	0.0011 (18)	−0.0016 (16)	−0.0044 (17)
C9	0.068 (2)	0.084 (2)	0.0632 (16)	−0.0165 (19)	0.0125 (16)	−0.0001 (16)
C11	0.063 (2)	0.094 (2)	0.0801 (19)	−0.0177 (19)	0.0167 (18)	−0.0068 (19)
C12	0.066 (2)	0.107 (2)	0.0689 (17)	−0.004 (2)	0.0148 (18)	−0.0094 (19)
C5	0.072 (2)	0.0706 (18)	0.0706 (17)	0.0124 (17)	−0.0058 (17)	−0.0094 (17)
N1	0.094 (2)	0.128 (3)	0.101 (2)	−0.030 (2)	−0.031 (2)	0.034 (2)

### Geometric parameters (Å, °)

**Table:**

O1—C6	1.365 (4)	C10—C11	1.371 (4)
O1—C7	1.403 (4)	C10—C9	1.377 (4)
F1—C1	1.350 (3)	C1—C2	1.359 (4)
C3—C4	1.379 (5)	C1—C6	1.380 (4)
C3—C2	1.391 (4)	C8—C9	1.378 (4)
C3—C13	1.425 (4)	C8—H8	0.9300
C13—N1	1.135 (4)	C6—C5	1.375 (4)
C7—C12	1.368 (5)	C2—H2	0.9300
C7—C8	1.368 (4)	C9—H9	0.9300
O2—C10	1.375 (3)	C11—C12	1.376 (4)
O2—H201	0.8200	C11—H11	0.9300
C4—C5	1.379 (4)	C12—H12	0.9300
C4—H4	0.9300	C5—H5	0.9300
			
C6—O1—C7	118.0 (2)	C9—C8—H8	120.0
C4—C3—C2	119.7 (3)	O1—C6—C5	124.6 (3)
C4—C3—C13	119.8 (3)	O1—C6—C1	116.9 (3)
C2—C3—C13	120.5 (3)	C5—C6—C1	118.4 (3)
N1—C13—C3	177.8 (5)	C1—C2—C3	118.4 (3)
C12—C7—C8	120.1 (3)	C1—C2—H2	120.8
C12—C7—O1	118.1 (3)	C3—C2—H2	120.8
C8—C7—O1	121.6 (3)	C10—C9—C8	119.9 (3)
C10—O2—H201	109.5	C10—C9—H9	120.0
C5—C4—C3	120.7 (3)	C8—C9—H9	120.0
C5—C4—H4	119.6	C10—C11—C12	119.7 (3)
C3—C4—H4	119.6	C10—C11—H11	120.1
C11—C10—O2	117.7 (3)	C12—C11—H11	120.1
C11—C10—C9	119.9 (3)	C7—C12—C11	120.3 (3)
O2—C10—C9	122.4 (3)	C7—C12—H12	119.8
F1—C1—C2	118.7 (3)	C11—C12—H12	119.8
F1—C1—C6	118.5 (3)	C6—C5—C4	119.9 (3)
C2—C1—C6	122.8 (3)	C6—C5—H5	120.0
C7—C8—C9	119.9 (3)	C4—C5—H5	120.0
C7—C8—H8	120.0		
			
C6—O1—C7—C12	−129.3 (3)	C4—C3—C2—C1	0.0 (5)
C6—O1—C7—C8	56.0 (4)	C13—C3—C2—C1	179.5 (3)
C2—C3—C4—C5	−0.7 (5)	C11—C10—C9—C8	0.8 (5)
C13—C3—C4—C5	179.8 (3)	O2—C10—C9—C8	−179.2 (3)
C12—C7—C8—C9	1.2 (5)	C7—C8—C9—C10	−1.6 (5)
O1—C7—C8—C9	175.8 (3)	O2—C10—C11—C12	−179.7 (3)
C7—O1—C6—C5	27.9 (4)	C9—C10—C11—C12	0.3 (5)
C7—O1—C6—C1	−155.4 (3)	C8—C7—C12—C11	−0.1 (5)
F1—C1—C6—O1	1.0 (4)	O1—C7—C12—C11	−174.8 (3)
C2—C1—C6—O1	−179.0 (3)	C10—C11—C12—C7	−0.7 (5)
F1—C1—C6—C5	177.9 (3)	O1—C6—C5—C4	178.0 (3)
C2—C1—C6—C5	−2.1 (5)	C1—C6—C5—C4	1.4 (5)
F1—C1—C2—C3	−178.6 (3)	C3—C4—C5—C6	0.0 (5)
C6—C1—C2—C3	1.4 (5)		

### Hydrogen-bond geometry (Å, °)

**Table:**

*D*—H···*A*	*D*—H	H···*A*	*D*···*A*	*D*—H···*A*
O2—H201···N1^i^	0.82	2.03	2.839 (4)	168

Symmetry codes: (i) −*x*+1/2, −*y*+2, *z*+1/2.

## References

[bb1] Bao, W. J., Wu, Y. G., Mao, C. H., Chen, M. & Huang, M. Z. (2007). *Fine Chem. Intermed.* **37**, 9–13.

[bb2] Farrugia, L. J. (1997). *J. Appl. Cryst.* **30**, 565.

[bb3] Farrugia, L. J. (1999). *J. Appl. Cryst.* **32**, 837–838.

[bb4] Higashi, T. (1995). *ABSCOR* Rigaku Corporation, Tokyo, Japan.

[bb5] Liu (2002). Please supply full reference.

[bb6] Luo, S., Zhang, J., Wang, J. & Li, B. (2009). *Acta Cryst.* E**65**, o2011. 10.1107/S1600536809029201 PMC2977414 21583682

[bb7] Rigaku (2006). *PROCESS-AUTO* Rigaku Corporation, Tokyo, Japan

[bb8] Rigaku/MSC (2007). *CrystalStructure* Rigaku/MSC, The Woodlands, Texas, USA.

[bb9] Sheldrick, G. M. (2008). *Acta Cryst.* A**64**, 112–122. 10.1107/S0108767307043930 18156677

[bb10] Sørensen, H. O. & Stuhr-Hansen, N. (2009). *Acta Cryst.* E**65**, o13. 10.1107/S1600536808039469 PMC2967934 21581591

[bb11] Zhang, J., Wu, J., Wang, J., Li, Y. & Luo, S. (2009). *Acta Cryst.* E**65**, o2340. 10.1107/S1600536809035247 PMC2970212 21577811

